# Beneficial Effects of Common Bean on Adiposity and Lipid Metabolism

**DOI:** 10.3390/nu9090998

**Published:** 2017-09-09

**Authors:** Henry J. Thompson, John N. McGinley, Elizabeth S. Neil, Mark A. Brick

**Affiliations:** 1 Cancer Prevention Laboratory, Colorado State University, Fort Collins, CO 80523, USA; john.mcginley@colostate.edu (J.N.M.); elizabeth.neil@colostate.edu (E.S.N.); 2 Department of Soil and Crop Sciences, Colorado State University, Fort Collins, CO 80523, USA; mark.brick@colostate.edu

**Keywords:** pulse crops, bean, adiposity, fatty acid oxidation

## Abstract

In developed countries which are at the epicenter of the obesity pandemic, pulse crop consumption is well below recommended levels. In a recent systematic review and meta-analysis of 21 randomized controlled clinical trials, pulse consumption was associated with improved weight control and reduced adiposity, although the underlying mechanisms were a matter of speculation. Common bean (*Phaseolus vulgaris* L.) is the most widely consumed pulse crop and was the focus of this investigation. Using outbred genetic models of dietary induced obesity resistance and of dietary induced obesity sensitivity in the rat, the impact of bean consumption was investigated on the efficiency with which consumed food was converted to body mass (food efficiency ratio), body fat accumulation, adipocyte morphometrics, and patterns of protein expression associated with lipid metabolism. Cooked whole bean as well as a commercially prepared cooked bean powders were evaluated. While bean consumption did not affect food efficiency ratio, bean reduced visceral adiposity and adipocyte size in both obesity sensitive and resistant rats. In liver, bean consumption increased carnitine palmitoyl transferase 1, which is the rate limiting step in long chain fatty acid oxidation and also resulted in lower levels of circulating triglycerides. Collectively, our results are consistent with the clinical finding that pulse consumption is anti-obesogenic and indicate that one mechanism by which cooked bean exerts its bioactivity is oxidation of long chain fatty acids.

## 1. Introduction

Pulse crops, i.e., grain legumes, are staple foods that are characteristic components of dietary patterns typically consumed in developing countries [1,2]. Among the 17 pulses identified as food sources by FAO, the four most widely produced are common bean (*Phaseolus vulgaris* L.), chickpea (*Cicer arietinum* L.), dry pea (*Pisum sativum* L.), and lentil (*Lens culinaris* L.) [3]. Pulses are concentrated sources of protein and dietary fiber and have very low fat content [1]. However, the consumption of pulses generally decreases as the economic status of a country improves and these grain legumes are displaced from the diet by animal products and refined protein sources such as soy which is derived from an oil seed legume [4]. While there are many economic and environmental arguments for maintaining the consumption of pulse crops as a primary source of dietary protein and fiber, only limited attention has been directed to the potential health benefits of retaining pulses as a primary food source in Western diets [4,5].

The occurrence of obesity is now considered to be a global pandemic and it is widely recognized that in addition to the metabolic disorders associated with obesity per se, e.g., non-alcoholic fatty liver disease, that obesity also increases the risk for a number of chronic diseases including type-2 diabetes, cardiovascular disease, and cancer [6]. Recently, a concerted effort was made to identify prospective clinical studies in which the effect of pulse crops on human health were investigated [7]. In the resulting systematic review and meta-analysis, a total of 21 randomized controlled trials were identified that investigated the effect of dietary pulse consumption on indicators of obesity including body weight, waist circumference, and body fat. Dietary pulses at a median dose of 132 g/day were found to lower body weight (mean difference = −0.34 kg (95% CI: −0.63 to −0.04 kg)) and body fat (mean difference = −0.34% (95% CI: −0.71 to 0.03)), with no effect on waist circumference. Dietary pulses lowered body weight significantly more than control diets under both weight maintaining and calorie restricted weight loss. The mechanistic basis advanced to explain these results included increased satiety attributed to the high fiber and protein content of pulses, the low glycemic index of pulses, and reduced bioavailability of calories from dietary pulses due to their content of dietary fiber. 

Of the four most prominent pulses, common bean is consumed in the largest amount per capita and was the focus of this investigation. A series of experiments were conducted to examine the effects of bean consumption on adiposity and lipid metabolism in a model system of polygenic obesity. This system was developed by divergent selection over 20 generations for animals with a high or low efficiency of converting ingested food to body mass (food efficiency ratio) in an outbred population of Sprague Dawley rats [8]. The selection process resulted in rats that were either dietary induced obesity sensitive (OS) or dietary induced obesity resistant (OR) when fed a diet containing 32% of calories from fat, a level of fat recommended for human consumption [5]. We used this model system to deconstruct the clinical observations regarding the anti-obesogenic activity of pulses and to evaluate the putative mechanisms purported to explain the protective activity of pulse crops.

## 2. Materials and Methods

### 2.1. Experimental Animals

Breeder pairs (approximately 30 pairs each Levin OR and OS rats were obtained from Taconic (Taconic, Hudson, NY, USA) at 5–7 weeks of age. These outbred strains of Sprague Dawley rats were originally obtained by Taconic from B.E. Levin after 20 generations of selective breeding for rapid weight gain on sucrose and moderate fat (32%) (SUMO32) [8]. These rats were subsequently outbred using a rotational breeding scheme for an additional 30+ generations, and were commercially available from the Taconic repository (strain: TacLevin:CD(SD)DIO, stock #DS; TacLevin:CD(SD)DR, stock #DR). In-house breeding was conducted using the Poiley rotational breeding scheme, in which breeder pairs are systematically rotated in each breeding cycle [9]. Pups were weaned at 3 weeks of age and were immediately switched to SUMO32 diet (Supplementary Table S1). Rats were maintained on a 12 h light/dark cycle at 24 ± 2 °C with 30% relative humidity, and given *ad libitum* access to SUMO32 diet and distilled water until they were randomized to experimental groups. All animal studies were performed in accordance with the Colorado State University Institutional Animal Care and Use Committee.

### 2.2. Experimental Design

#### 2.2.1. Experiments 1 and 2

For Experiment 1, 25 twenty-eight day old female rats from our OS breeding colony were randomized to one of two diet groups and fed either control diet or diet to which cooked, whole bean had been added. Diet formulations are detailed in Supplementary Tables S1 and S2. Rats were fed *ad libitum* and the experiment was terminated after 26 days of feeding these diets. In Experiment 2, 26 twenty-eight day old OS female rats were used. The only difference between Experiments 1 and 2 was that cooked, processed bean powder was used in Experiment 2 instead of whole cooked bean powder. In addition, the study was terminated 29 days after initiation of experimental diet feeding. In both experiments, rats were group housed 3 per cage.

#### 2.2.2. Experiment 3

In this experiment, both OS and OR rats from our breeding colony were used. Rats were group housed during the day from 9 a.m. to 4 p.m. without access to food. For the overnight period (dark cycle) the rats were transferred to individual housing in wire mesh bottomed cages equipped with tunnel feeders so that food intake could be accurately quantified. Fresh pre-measured food was given every overnight feeding period. Rats were allowed to acclimate to the caging system and feeding approach for 9 days after which the study commenced. Prior to study initiation, rats were paired by body weight and pairs were randomized to either the control diet or the bean containing diet. There were 7 pairs of OS rats and 6 pairs of OR rats. Each bean fed rat was given *ad libitum* access to pre-measured food. Within a pair, the amount of food consumed by the bean fed rat was given to its paired control over the subsequent 24 h period. This process was repeated for the 25 day duration of the study. 

### 2.3. Experimental Diets

A purified diet formulation, sucrose and moderate 32% fat diet (SUMO32) is described in detail in Supplementary Table S1. The experimental diets were a modification of SUMO32. Uncooked white kidney (Cannellini) bean was provided by Archer Daniels Midland Company (Decatur, IL, USA) and was sent to Bush Brothers & Company (Chestnut Hill, TN, USA) for canning. Cooked whole beans were packed in standard brine without the incorporation of any additives. The canned, cooked beans were then sent to Van Drunen Farms, (Momence, IL, USA) where the beans were drained and freeze dried. The freeze dried bean was then milled into a homogeneous powder and sent to our laboratory and stored at −20 °C until incorporated into diets. As an alternative source of bean, commercially prepared cooked bean powder was provided by Archer Daniels Midland Company (Decatur, IL, USA). Four bean market classes; Black, Navy, Great Northern, and Pinto bean were combined in equal parts to make up the bean powder added to the modified SUMO32 diet formulation. Diets were formulated using specific guidelines [10] and adjusted using the proximate analysis of the cooked whole bean powder (IEH-Warren Laboratory, Greeley, CO, USA) and the proximate data provided by the vendor of the commercially processed bean powder (Archer Daniels Midland Company (Decatur, IL, USA)). The proximate analysis data are shown in Supplementary Table S2. 

### 2.4. Necropsy

Rats were anesthetized using isoflurane inhalation. Blood was obtained by cardiac puncture prior to the rat being euthanized by cervical dislocation. Blood was collected into serum separator tubes (SST) Vacutainer (Becton-Dickinson, Franklin Lakes, NJ, USA) allowed to clot at room temperature for 30 min prior to centrifugation. Serum was kept on ice and then stored at −80 °C until use. Livers were immediately removed after blood collection, freeze clamped, snap-frozen in liquid nitrogen, and stored at −80 °C. Visceral fat depots were harvested, weighed and a portion fixed in 10% neutral buffered formalin for histological evaluation. 

### 2.5. Western Blot-Based Immuno-Nanocapillary Electrophoresis

Samples were prepared and assayed according to a protocol previously published by our laboratory [11]. Briefly, frozen tissues were removed from −80 °C storage and quickly ground to a fine powder in liquid nitrogen using ceramic mortars and pestles. Lysis buffer was prepared using ice cold T-PER tissue protein extraction reagent (Thermo Fisher Scientific, Waltham, MA, USA) with Halt protease and phosphatase inhibitor cocktail at 1:50 (Thermo Fisher Scientific), and 0.5M EDTA at 1:100 to inhibit metalloproteases. Ice cold 7.5 mL glass Dounce homogenizers (VWR, Radnor, PA, USA) were filled with 1.5 mL of lysis buffer and tared on the scale. Frozen tissue powder was quickly weighed in the Dounce and then homogenized on ice using 10 full strokes of the glass pestle followed by vortexing for 5 s and sonication (Branson Sonifier S-250A, Fisher Scientific), using 1 pulse (10% duty cycle and control set at 5). The homogenization process was repeated 4 times and samples were allowed to sit on ice for an additional 10 min prior to transfer to 2.0 mL microfuge tubes and centrifugation at 12,000× *g* for 20 min. A glass Pasteur pipet was used to extract the supernatant sandwiched between the cell pellet and surface lipid layer. The lysate supernatant was divided into 25 µL single use aliquots using 0.2 mL PCR tubes and stored at −80 °C. Protein concentration was determined using the Bradford assay. Nanocapillary electrophoresis was performed using the WES instrument and proprietary kits (ProteinSimple, San Jose, CA, USA). The kits consisted of microplates with a prefilled section containing proprietary reagents, cartridge with 25 nano capillaries, lyophilized standard pack (DTT, biotinylated ladder and fluorescent standards), 10× sample buffer, secondary antibody, HRP-conjugated streptavidin (ladder only), luminol-S, peroxide, antibody diluent and wash buffer. Briefly, samples were prepared by adding 2.5 µL of diluted tissue lysate to a 0.2 mL PCR tube containing 1.5 µL of 5× fluorescent master mix and 3.5 µL of 0.1× sample buffer. Samples were denatured in a dry bath at 95 °C for 5 min. Samples, biotinylated ladder, multiplexed primary antibodies, HRP-conjugated secondary antibody, chemiluminescent substrate (luminol-S peroxide) and wash buffer were pipetted into the appropriate wells according to kit instructions. The capillary cartridge and microplate were loaded into the fully automated WES instrument. The entire assay was completed within each capillary as follows: the vacuum manifold loaded each capillary with a separation matrix, stacking matrix and sample; a voltage of 375 volts was applied for 30 min to separate the proteins based on size followed by exposure to UV light in order to immobilize the proteins in the capillary prior to immuno-labeling and subsequent detection resulting in the chemiluminescent signal intensity of each target protein displayed as an electropherogram. Each sample capillary was probed with the primary antibody for the protein of interest and an appropriate antibody used as a loading control, e.g., Vinculin (p-ACC^Ser79^, ACC and UCP1); DJ-1 (ACADL and ASCL4); and GAPDH (p-AMPK^Thr172^, AMPK, CD36 and CPT1). All values reported are normalized to loading controls. An electropherogram for each antibody used in this study, showing the peak that was quantified, determined on a pooled sample of 10 tissues as part of our quality control protocol for antibody selection, is shown along with the vendor and catalog number for each antibody in Supplementary Figure S1 and Supplementary Table S3. We also show a representative electropherogram in lane view, which is a virtual western blot-like image generated by the Compass software, version 3.1.7 (ProteinSimple, San Jose, CA, USA) that displays both the quantified band for CPT1 and the loading control (GAPDH) band to which it is normalized. Images of the electropherograms for each protein assessed are in Supplementary Figure S1.

### 2.6. Triglyceride Analysis

Triglyceride in serum was determined enzymatically using a commercially available kit (Pointe Scientific, Inc., Canton, MI, USA). Triglyceride in liver was determined enzymatically using a commercially available kit (Cayman Chemical, Ann Arbor, MI, USA).

### 2.7. Morphometric Analysis of Adipose Tissue

Images of H&E stained adipose tissue were captured at 100× magnification using a Zeiss AxioCamHR (Zeiss, Thornwood, NY, USA) digital camera mounted on a Zeiss Axioskop II microscope. Prior to capturing images, background shading correction was achieved using the Zeiss AxioVision software (Zeiss, Thornwood, NY, USA) (Representative image, Supplementary Figure S2). Images were captured (1300 × 1030 pixels, 24 bit RGB, 150 DPI) and saved as JPEG files. A macro was written in Image Pro Plus version 4.5 (Media Cybernetics, Inc., Rockville, MD, USA) to facilitate analysis. The macro was designed to load a calibration data file, one color JPEG image at a time and then convert the image to 8-bit grayscale (256 shades of gray). Once converted, the macro applied a predefined look up table to enhance image brightness and contrast. In addition, morphological filters were applied to enhance adipocyte borders. This pre-processing resulted in a black and white image suitable for analysis, e.g., black adipocyte borders on a white background. The segmentation range was set 10–255, i.e., black adipocyte borders (range 0–9) were excluded and only the white vacuolated spaces (adipocytes) would be included in the analysis. The clean borders option was enabled so that partially visible adipocytes at the periphery of the image were excluded. The minimum area was set to 131 µm^2^ to eliminate any small objects that were not adipocytes. The software filled the remaining area objects with red making it easier for the user to visualize. The macro paused allowing the user to toggle any of these objects on or off prior to measurement, e.g., a large white space created as a result of cutting artifact. Once the user continued execution of the macro all area objects within specified parameters were counted and measured automatically. Measurements for each object were then exported automatically via dynamic data exchange to an Excel spreadsheet for analysis. 

### 2.8. Statistical Analyses

The data collected in this study were found to be normally distributed. Data from Experiments 1 and 2 were evaluated by analysis of variance. Experiment 3 data were evaluated by factorial analysis of variance and relationships among variables were also evaluated by regression analysis. Data analysis was conducting using Systat version 13.0 (Systat Software, Inc., San Jose, CA, USA) and GraphPad Prism, version 5.2 (GraphPad Software, Inc., La Jolla, CA, USA).

## 3. Results

### 3.1. Effect of Bean on Body Weight and Visceral Fat Deposition under Ad Libitum Feeding Conditions

Two ad libitum feeding studies were conducted. In the first study, cooked whole bean was fed so that 75% of dietary protein was provided by bean and the remainder by casein, an animal based protein source. As shown in Table 1, bean consumption resulted in rats that were 10.5% lighter. Visceral adipose depots were normalized to tibia length to adjust for differences in body weight as is commonly done in rodent obesity studies [12]. There was an overall difference of 65.3% between total visceral adiposity in rats fed control versus bean containing diet. The greatest reductions in fat pad mass were observed in the parametrial and retroperitoneal fat pads. 

Since there is a growing trend to increase bean consumption via its incorporation into various food products, commercially available bean powders were obtained and incorporated into the diet at the same level as the cooked whole bean. The response of the rats to bean consumption was remarkably similar (Table 2). There was a 13.5% difference in body weight of the rats and visceral adiposity differed by 42.7%.

### 3.2. Effect of Bean on Feed Efficiency, Visceral Fat Disposition, and Hepatic Lipid Metabolism under Paired-Feeding Conditions

The ad libitum feeding approach used in the first two experiments indicated that dietary bean had effects on palatability and/or satiety that impacted weight gain, but such effects could be explained by differences in the bioavailability of calories from a bean containing diet. To evaluate this possibility, we used the commercially available bean powder in a paired-feeding study and evaluated how bean feeding affected the conversion of consumed food to body mass (food efficiency ratio). As shown in Table 3 and contrary to the suggestion of differences in energy bioavailability, the food efficiency ratio was the same for control and bean fed rats whether they were obesity sensitive or obesity resistant. However, despite the fact that both groups had the same weight gain and final body weight, the bean fed rats had reduced visceral adiposity. The magnitude of the difference in total visceral adiposity between control and bean fed rats was 27.8% in the OS rat strain and 43.4% in the OR rat strain. While bean consumption reduced the mass of the three visceral fat pads that were measured, the parametrial and perirenal fat pads were reduced in mass to the greatest extent.

**Figure 1:**
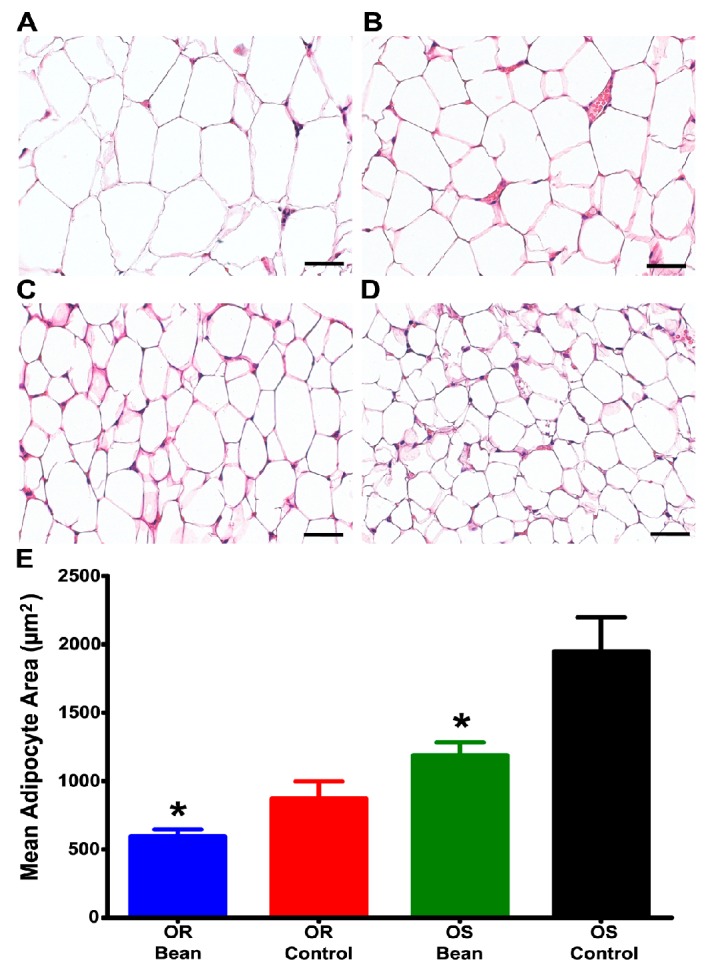
Adipocyte image analysis of H&E stained parametrial fat. (**A**) OS Control *n* = 7; (**B**) OS Bean *n* = 7; (**C**) OR Control *n* = 6; (**D**) OR Bean *n* = 6; (**E**) Graph of adipocyte image analysis results. Factorial ANOVA: strain *p* = 0.0001, diet *p* = 0.002, interaction *p* = 0.112; * different than respective control (*p* < 0.05). Images are shown at 400× magnification for clarity, but analysis was done at 100× magnification, bars = 40 µM.

To further examine the nature of this effect, we evaluated adipocyte morphmetrics in the parametrial fat pad. As shown in Figure 1, bean fed rats had smaller adipocytes in both OS and OR rats.

### 3.3. Investigation of Mechanisms

Tissue from Experiment 3 was used for mechanistic studies since the effects of bean feeding were independent of differences in body weight gain and food efficiency. A focus on fatty acid oxidation in liver was prompted by reports that differences in fatty acid oxidation in OR versus OS rats accounted for resistance versus sensitivity to obesity in these rats strains [13,14]. Table 4 summarizes the analyses that were performed using a western blot based immuno-nanocapillary electrophoresis system to quantify differences in protein expression. Four proteins involved in fatty acid oxidation were assessed, acyl CoA synthase (long chain fatty acid isoform 4) (ACSL4), acyl CoA dehydrogenase for long chain fatty acids (ACADL), fatty acid translocase (CD36), and carnitine palmitoyl transferase 1 (CPT1). Bean consumption increased expression of all four proteins with the difference in CPT1 being statistically significant (Table 4). These same data were subjected to multivariate analysis of variance to assess if the overall capacity for β-oxidation was increased by bean feeding. With adjustment of the model for effects due to animal pairings and differences in food efficiency among pairs, the Hotelling statistic was highly significant for an overall increase in β-oxidation associated with bean feeding (*p* = 0.001). Focusing on CPT1 which is the rate limiting step in β-oxidation and that was significantly different between control and bean fed rats, proteins that regulate CPT1 expression were assessed. Amount of acetyl CoA carboxylase (ACC) and ^Ser79^pACC (which is inactive) were significantly lower in bean fed rats, a finding consistent with CPT1 induction. Since ACC is regulated by AMP activated protein kinase (AMPK), we also assessed its activity and it was higher in bean fed rats, but the difference from control rats was not statistically significant. Antibody quality control data are presented in Supplementary Figure S1.

**Figure 2:**
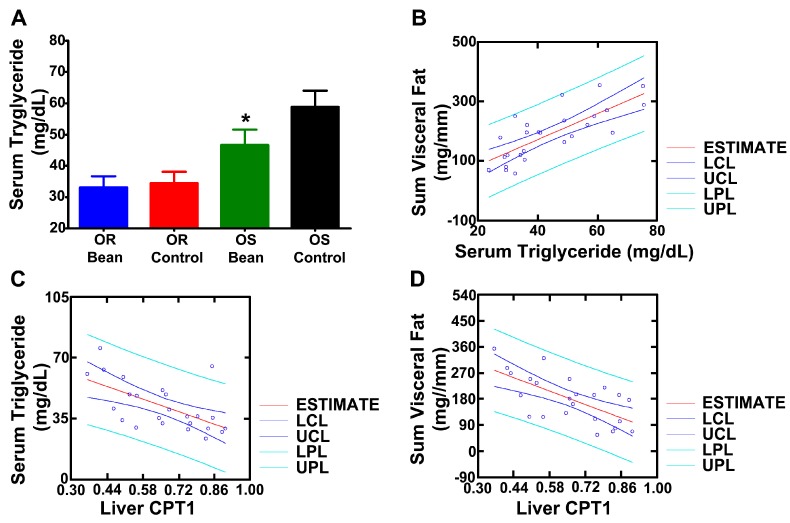
Effects on lipid metabolism (**A**) Serum triglycerides; different than respective control, *p* < 0.05); (**B**) Regression of sum visceral fat vs. serum triglyceride; (**C**) Regression of serum triglyceride vs. liver CPT1 expression; (**D**) Regression of sum visceral fat to liver CPT1 expression. LCL: lower confidence limit; UCL: upper confidence limit; LPL: lower prediction limit; UPL: upper prediction limit.

We predicted that serum triglycerides would be reduced by bean feeding [15]. As shown in Figure 2A, circulating triglyceride was lower in both bean fed groups and was significantly different from control in the OS rats and circulating triglyceride was positively associated with visceral adiposity (Figure 2B, *r* = 0.729, *p* = 0.0002). CPT1 is a rate limiting step in β-oxidation and has been suggested to account for difference in obesity sensitivity. To assess this idea, a series of regression analyses were performed. CPT1, which bean feeding induced, was negatively correlated with plasma triglyceride (Figure 2C, *r* = −604, *p* = 0.005) and with total visceral adiposity (Figure 2D, *r* = −656, *p* = 0.002). We also assessed liver triglyceride content (Table 5) and found the same pattern of reduction in response to bean feeding as observed with serum triglyceride and hepatic CPT1.

## 4. Discussion

Pulse consumption is associated with a modest, albeit significant, reduction in body weight as recently reported in a systematic review and meta-analysis of 21 prospective trials in which the clinical impact of pulse consumption was assessed [7]. Given the complexity of body weight regulation, many factors could account for the activity of pulses. Therefore, the study reported herein was initiated in which preclinical models of resistance and sensitivity to obesity were used to deconstruct the clinical observations. Experiments were conducted using either cooked whole bean powder or a commercially prepared cooked bean powder that is being used to develop new food products. Ad libitum feeding was used to model mechanisms related to satiety inducing (anorexigenic) effects of bean since rats are known to eat to meet caloric needs. A paired-feeding technique in which control rats were fed the amount of food consumed by their bean-fed paired counterparts was used to investigate effects on the food efficiency ratio.

Ad libitum consumption of bean from either dietary source resulted in less weight gain and reduced storage of lipid in visceral fat depots (Table 1 and Table 2). Relative to the magnitude of difference in weight gain observed between control and bean-fed rats (approximately 10 to 14%), the accumulation of lipid in the visceral fat depots differed to a much greater extent (40 to 60%) indicating a selective effect of bean on metabolism that led to a marked reduction in visceral adiposity. The importance of this effect is derived, in part, from evidence that visceral adipose storage sites are an origin of the metabolic dysfunction associated with obesity [16]. Thus, in this regard, the bioactivity resulting from bean consumption is anti-obesogenic. However, further evidence was sought to determine whether the anti-obesogenic activity of bean was independent of differences in body weight gain. A paired-feeding technique, in which food intake was held constant between rats fed either the bean containing or the control diet (Experiment 3), was used to examine this issue [17].

By design, there was no difference in food consumption between control and bean fed rats using the paired feeding technique. Under these conditions, the efficiency with which ingested food was converted to body mass did not differ between diet treatments; however, visceral adiposity (Table 3) was markedly reduced in bean-fed rats. These findings establish that bean consumption has an anti-obesogenic effect independent of food intake or body weight gain and extend the observations to adipocyte size (Figure 1). The fact that a significant reduction in adipocyte size was observed in both OR and OS bean fed animals is consistent with reduced risk of metabolic syndrome [16]. Moreover, these data fail to support the commonly cited idea that the anti-obesogenic activity of bean is dependent on a reduction in the bioavailability of calories from bean containing diet due to its high content of dietary fiber [7]. This point was critically tested by our inclusion of both OR and OS rats in this experiment. While there was a 35% difference in the food efficiency ratio between OR and OS rats fed the control diet, bean feeding had no effect on this ratio in either rat strain yet there was a highly significant reduction in visceral adipose mass in both OR and OS rats in response to bean feeding. The finding of this effect in both OS and OR rats also suggests that bean consumption would benefit people in controlling their body weight and in maintaining a healthy metabolic profile across a wide range of body mass indices including normal weight as well as overweight and obese individuals. 

An advantage of using the OR and OS strains is that it has been reported that OR rats have a higher rate of long chain fatty acid oxidation in the liver than OS rats. Increased fatty acid oxidation in OR rats has been suggested to account at least in part for the difference in adiposity between the two strains [13,14]. Consistent with other reports [13,14], we observed increased levels of carnitine palmitoyl transferase I (CPT1) in OR versus OS rats fed the control diet. CPT1 catalyses the transport of long chain fatty acids across the inner mitochondrial membrane, which is the rate limiting step in the β-oxidation of long chain fatty acids. It is noteworthy that there is a strong correlation between CPT1 expression and rates of long chain fatty acid oxidation measured radiometrically [13,14]. As reported in Table 4, both OR and OS bean-fed rats had significantly higher levels of CPT1 than their respective control fed rats. Other enzymes that have been found to be associated with the differences in obesity sensitivity in the OS versus OR rat strains and that are involved in β-oxidation are acyl CoA dehydrogenase and acyl CoA synthase. Higher levels of both enzymes as well as CD36, a fatty acid translocase that transports lipids into the cell, were observed in bean fed rats. To assess whether CPT1 is mediating the effects of bean on visceral adiposity, a series of regression analyses were performed (Figure 2). CPT1, which bean feeding induced, was negatively correlated with total visceral adiposity and plasma triglyceride and circulating triglyceride was positively associated with visceral adiposity. These findings supports the likelihood of a causal link between the effect of bean feeding on fatty acid oxidation and the observed reduction in visceral adipose mass in bean fed rats in both OR and OS strains. 

Since the transfer of long chain fatty acids across the inner mitochondrial membrane by CPT1 is rate limiting, and CPT1 is inhibited by malonyl CoA, the product of acetyl CoA carboxylase, we accessed the amount of ACC and its phosphorylated formed which is inactive. However, the phosphorylation data for ACC as well as the AMPK indicate that regulation of ACC in bean fed rats is likely due to effects on a transcription factor(s) rather than via an alteration in ACC activity mediated by AMPK. Candidate transcription factors that regulate fatty acid oxidation include peroxisome proliferator-activated receptors (PPARs) and mammalian target of rapamycin (mTOR). An alternative mechanism by which bean could affect fat utilization in adipose tissue is via uncoupling of oxidative phosphorylation; however, bean consumption did not affect UCP1 expression in visceral adipose tissue (Supplementary Figure S3), and therefore this line of investigation was not further pursued. 

There are a number of reports in the literature about the effects of extracts of raw bean on various aspects of weight control (reviewed in [18]) and it is important to note that the effects reported herein are unrelated to those findings. While raw bean contains α-amylase inhibitors and phytohemoagglutinins (lectins) which have been reported to affect body weight regulation, this study was conducted using cooked bean powders in which these proteins are not detectable because they are heat inactivated. Thus, the effects reported are highly relevant in using a food source that is high in protein, fiber, and many small molecules, e.g., polyphenols, with essentially no lipid content. While we have observed an anti-obesogenic effect at levels of bean consumption in rodents (20% *w*/*w* in a purified diet), which are similar to the median human intake of 132 g pulses per day as reported in [19], this study was designed to investigate the effects cooked bean consumption when 75% of dietary protein was provided from a plant source and the remaining 25% from an animal source of protein, a ratio consistent with WHO sustainability and global food security goals [20,21].

## 5. Conclusions

Pulses such as common bean are neglected staple food crops in many Western societies that are experiencing a pandemic increase the in prevalence of obesity [22,23]. The WHO sponsored “Year of the Pulse” promoted renewed interest in this highly available and affordable food source [24]. The data reported herein indicate that common bean has specific anti-obesogenic activity which could lessen the impact of obesity on chronic diseases in individuals who are already overweight or obese and potentially reduce the risk of adult weight gain by inhibiting accumulation of lipid in visceral fat depots. Additional work is required to establish the specific mechanisms that account for the effects of common bean on lipid metabolism and to determine if the anti-obesogenic activity of common bean is found in all pulse crops.

## Figures and Tables

**Table 1:** Effect of ad libitum feeding of cooked whole bean diet on weight gain and visceral adiposity.

Diet	Initial Body Weight ^1^ (g)	Final Body Weight (g)	Weight Gain/d (g)	Tibia Length (mm)	Retro-Peritoneal Fat ^2^ (mg/mm)	Para-Metrial Fat (mg/mm)	Peri-Renal Fat (mg/mm)	Total Visceral Fat (mg/mm)
Control	65.0 ± 2.2	164.2 ± 4.6	3.8 ± 0.1	28.5 ± 0.3	21.8 ± 2.0	31.4 ± 2.7	7.0 ± 0.6	60.1 ± 4.8
Bean	62.2 ± 2.6	147.7 ± 4.8	3.3 ± 0.1	27.5 ± 0.3	10.5 ± 0.7	15.8 ± 1.2	4.3 ± 0.3	30.5 ± 1.9
*p*-value	0.43	0.02	0.001	0.019	7 × 10^−5^	7 × 10^−5^	0.001	3 × 10^−5^

^1^ Values are means ± SEM; ^2^ Units are mg mass divided by length of tibia in mm; Control *n* = 13, Bean *n* = 12.

**Table 2:** Effect of ad libitum feeding of commercially processed bean powder on weight gain and visceral adiposity.

Diet	Initial Body Weight ^1^ (g)	Final Body Weight (g)	Weight Gain/day (g)	Tibia Length (mm)	Retro-Peritoneal Fat ^2^ (mg/mm)	Para-Metrial Fat (mg/mm)	Peri-Renal Fat (mg/mm)	Total Visceral Fat (mg/mm)
Control	66.6 ± 2.4	187.2 ± 4.0	4.2 ± 0.1	29.7 ± 0.3	27.1 ± 2.6	45.5 ± 4.9	12.3 ± 1.2	84.9 ± 7.7
Bean	66.8 ± 2.5	163.6 ± 3.2	3.3 ± 0.1	29.0 ± 0.3	17.3 ± 1.3	30.4 ± 3.1	7.4 ± 0.7	55.1 ± 4.7
*p*-value	0.95	1.3 × 10^−4^	2 × 10^−5^	0.107	0.003	0.017	0.002	0.004

^1^ Values are means ± SEM; ^2^ Units are mg mass divided by length of tibia in mm; Control *n* = 13, Bean *n* = 13.

**Table 3:** Effect of paired-feeding of commercially processed bean powder on food efficiency ratio and visceral adiposity.

Diet ^1^	Final Body Weight ^1^ (g)	Feed Efficiency Ratio (g)	Retro-Peritoneal Fat ^2^ (mg/mm)	Para-Metrial Fat (mg/mm)	Peri-Renal Fat (mg/mm)	Total Visceral Fat (mg/mm)
	**Obesity Sensitive (OS)**					
Control	230 ± 3	0.321 ± 0.008	77.1 ± 6.8	185.6 ± 14.7	25.9 ± 2.3	288.6 ± 22.3
Bean	226 ± 4	0.323 ± 0.006	67.5 ± 5.2	136.9 ± 5.3	13.8 ± 1.9	218.2 ± 9.4
	**Obesity Resistant (OR)**					
Control	166 ± 4	0.251 ± 0.005	38.5 ± 4.4	88.2 ± 9.3	14.2 ± 0.8	140.8 ± 12.7
Bean	164 ± 4	0.260 ± 0.006	25.1 ± 4.3	59.9 ± 11.2	5.7 ± 0.8	90.7 ± 16.0
	**Factorial Analysis of Variance (*p*-Values) ^3^**					
Strain	1.8 × 10^−11^	1.3 × 10^−9^	2.1 × 10^−7^	5.1 × 10^−8^	1.0 × 10^−5^	2.1 × 10^−8^
Diet	0.463	0.394	0.047	0.002	4.8 × 10^−6^	0.001
Interaction	0.837	0.571	0.734	0.354	0.309	0.537

^1^ Values are means ± SEM; ^2^ Units are mg mass divided by length of tibia in mm; ^3^ For factorial ANOVA, Strain is OR vs. OS, Diet is Control vs. Bean; OS Control *n* = 7, OS Bean *n* = 7, OR Control *n* = 6, OR Bean *n* = 6.

**Table 4:** Protein expression in liver.

	Obesity Sensitive (OS)		Obesity Resistant (OR)		*p*-Values ^2^		
Protein ^1^	Control	Bean	Control	Bean	Strain	Diet	Interaction
^Ser79^pACC	1.32 ± 0.07	1.02 ± 0.10	1.08 ± 0.09	0.67 ± 0.04	0.001	2.2 × 10^−4^	0.486
ACC	1.17 ± 0.08	0.73 ± 0.05	0.81 ± 0.06	0.66 ± 0.11	0.014	0.001	0.083
ACC Ratio	1.14 ± 0.06	1.40 ± 0.08	1.34 ± 0.09	1.12 ± 0.13	0.671	0.839	0.018
ACADL	7.00 ± 0.50	7.91 ± 0.44	5.54 ± 0.20	6.08 ± 0.29	3.2 × 10^−4^	0.070	0.630
ACSL4	1.49 ± 0.15	1.68 ± 0.03	1.12 ± 0.06	1.19 ± 0.06	4.0 × 10^−5^	0.119	0.468
^Thr172^pAMPK	0.93 ± 0.06	0.95 ± 0.08	0.95 ± 0.07	1.17 ± 0.10	0.144	0.137	0.245
AMPK	2.06 ± 0.10	2.25 ± 0.07	1.95 ± 0.06	2.27 ± 0.13	0.613	0.012	0.490
AMPK Ratio	0.46 ± 0.05	0.43 ± 0.04	0.49 ± 0.03	0.52 ± 0.04	0.162	0.969	0.452
CD36	1.27 ± 0.19	1.87 ± 0.50	1.53 ± 0.13	1.69 ± 0.33	0.912	0.273	0.523
CPT1	0.50 ± 0.05	0.67 ± 0.06	0.67 ± 0.06	0.81 ± 0.03	0.007	0.006	0.753

^1^ Values are peak heights normalized to a loading control, mean ± SEM; ^2^ For factorial ANOVA; Strain is OR vs. OS, Diet is Control vs. Bean; OS Control *n* = 7, OS Bean *n* = 7, OR Control *n* = 6, OR Bean *n* = 6.

**Table 5:** Effect of paired feeding of commercially processed bean powder on liver triglyceride.

Diet ^1^	Liver Triglyceride (mg/mg Protein × 10^−2^) ^2^
	**Obesity Sensitive (OS)**
Control	3.8 ± 0.5
Bean	2.4 ± 0.3
	**Obesity Resistant (OR)**
Control	3.4 ± 0.2
Bean	3.2 ± 0.2
	**Factorial Analysis of Variance (*p*-Values) ^3^**
Strain	0.541
Diet	0.036
Interaction	0.076

^1^ Values are means ± SEM; ^2^ Units are mg liver triglyceride per mg total protein × 10^−2^; ^3^ For factorial ANOVA, Strain is OR vs. OS, Diet is Control vs. Bean. OS Control *n* = 7, OS Bean *n* = 7, OR Control *n* = 6, OR Bean *n* = 6.

## Supplementary Materials

The following are available online at www.mdpi.com/2072-6643/9/9/998/s1, Table S1: Composition of Experimental Diets, Table S2: Proximate Data for Bean Powders, Table S3: Antibody List, Figure S1: Antibody quality control data, Figure S2: Adipocyte image analysis, Figure S3: UCP1 expression in visceral fat.

## Abbreviations

The following abbreviations are used in the manuscript:

ACC	Acetyl CoA carboxylase
ACADL	Acyl CoA dehydrogenase
ACSL4	Acyl CoA synthetase
AMPK	Adenosine monophosphate-activated protein kinase
CD36	Cluster of differentiation 36 (fatty acid translocase)
CPT1	Carnitine palmitoyl transferase I
mTOR	Mammalian target of rapamycin
PPAR	Peroxisome proliferator-activated receptor
OR	Obesity resistant
OS	Obesity sensitive
SUMO32	Sucrose and Moderate Fat 32% Diet
UCP1	Uncoupling Protein 1
