# Supplementary material for: Beneficial Effects of Common Bean on Adiposity and Lipid Metabolism
Source: Nutrients. 2017 Sep 9;9(9):998. doi: 10.3390/nu9090998 (PMC5622758; doi:10.3390/nu9090998)
Supplement: Supplementary file 1 [file nutrients-09-00998-s001.zip › Supplementary Table S1-Composition of Experimental Diets.docx]

**Supplementary Table S1.** Composition of Experimental Diets.

| **Ingredient** | **SUMO32 ^1^**  **(g/100g)** | **Control Diet ^2^**  **(g/100g)** | **Cooked, Whole Bean, Cannellini ^2^ (g/100g)** | **Cooked, Processed Bean, Market Basket ^2^**  **(g/100g)** |
| --- | --- | --- | --- | --- |
| Solka-Floc | 2.9 | 7.5 | 0.0 | 0.0 |
| Cannellini bean | 0.0 | 0.0 | 60.0 | 0.0 |
| Black bean | 0.0 | 0.0 | 0.0 | 15.0 |
| Pinto bean | 0.0 | 0.0 | 0.0 | 15.0 |
| Navy bean | 0.0 | 0.0 | 0.0 | 15.0 |
| Great Northern bean | 0.0 | 0.0 | 0.0 | 15.0 |
| Casein (>=85% protein) | 18.2 | 18.2 | 4.4 | 4.4 |
| Cerelose (Dextrose) | 7.2 | 7.2 | 1.6 | 1.6 |
| Corn Starch | 20.6 | 16.0 | 4.6 | 4.6 |
| Sucrose | 27.8 | 27.8 | 6.1 | 6.1 |
| Vitamin mix (AIN-93-VX) | 1.1 | 1.1 | 1.1 | 1.1 |
| DL-Methinonie | 0.3 | 0.3 | 0.3 | 0.3 |
| L-Tryptophan (Sigma T0254-25G) | 0.0 | 0.0 | 0.0 | 0.0 |
| Choline bitartrate (41% choline) | 0.2 | 0.2 | 0.2 | 0.2 |
| Mineral mix (AIN-93G-MX) | 3.8 | 3.8 | 3.8 | 3.8 |
| Calcium Carbonate | 0.5 | 0.5 | 0.5 | 0.5 |
| Sodium Bicarbonate | 0.5 | 0.5 | 0.5 | 0.5 |
| Potassium Citrate, 1 H_2_0 | 1.3 | 1.3 | 1.3 | 1.3 |
| Corn Oil | 11.3 | 11.3 | 11.3 | 11.3 |
| Butter Fat | 4.2 | 4.2 | 4.2 | 4.2 |
| TOTAL (g) | 100.0 | 100.0 | 100.0 | 100.0 |

^1^ Original SUMO32 diet formulation. ^2^ Experimental diets modified from the original SUMO32 diet formulation.
