# Supplementary material for: Beneficial Effects of Common Bean on Adiposity and Lipid Metabolism
Source: Nutrients. 2017 Sep 9;9(9):998. doi: 10.3390/nu9090998 (PMC5622758; doi:10.3390/nu9090998)
Supplement: Supplementary file 1 [file nutrients-09-00998-s001.zip › Supplementary Table S2- Proximate Data of Bean Powders.docx]

**Supplementary Table S2.** Proximate Analysis of Cooked Whole Bean and Cooked Processed Bean powders.

|  | **Cooked, Whole Cannellini Bean Powder (g/100g)** | **Cooked, Coomerically Processed, Market Basket Bean Powder ^1^ (g/100g)** |
| --- | --- | --- |
| Moisture | 10.6 | 9.5 |
| Protein | 22.2 | 21.8 |
| Crude Fat | 1.6 | 3.0 |
| Crude Fiber ^1^ | 22.0 | 24.3 |
| Nitrogen-Free Extract ^1^ | 38.9 | 38.3 |
| Ash | 3.7 | 3.3 |
| Total (g) | 100.0 | 100.0 |

^1^ Archer, Daniels Midland provided commercially available bean powders representing 4 common bean market classes: black, great northern, navy, and pinto. They were mixed in equal proportions to constitute the Market Basket bean powder.
