# Supplementary material for: Beneficial Effects of Common Bean on Adiposity and Lipid Metabolism
Source: Nutrients. 2017 Sep 9;9(9):998. doi: 10.3390/nu9090998 (PMC5622758; doi:10.3390/nu9090998)
Supplement: Supplementary file 1 [file nutrients-09-00998-s001.zip › Supplementary Table S3- Antibody List.docx]

**Supplementary Table S3**. Antibody List

| Primary Antibody | Vendor | Catalog # |
| --- | --- | --- |
| ^Ser79^pACC | Cell Signaling | 3661 |
| ACC | Cell Signaling | 3676 |
| ACADL | Aviva Systems Biology | arp33855_p050 |
| ACSL4 | Santa Cruz Biotechnology | sc-365230 |
| ^Thr172^pAMPK | Cell Signaling | 2535 |
| AMPK | Cell Signaling | 2603 |
| CD36 | Novus Biologicals | NB400-144 |
| CPT1 | Santa Cruz Biotechnology | sc-393070 |
| UCP1 | Abcam | AB23841 |
| Loading Controls | | |
| DJ-1 | Cell Signaling | 5933 |
| GAPDH | Santa Cruz Biotechnology | sc-25778 |
| Vinculin | Cell Signaling | 13901 |
