# Supplementary material for: Beneficial Effects of Common Bean on Adiposity and Lipid Metabolism
Source: Nutrients. 2017 Sep 9;9(9):998. doi: 10.3390/nu9090998 (PMC5622758; doi:10.3390/nu9090998)
Supplement: Supplementary file 1 [file nutrients-09-00998-s001.zip › Supplementary Figure S1-Antibody Quality Control Data.docx]

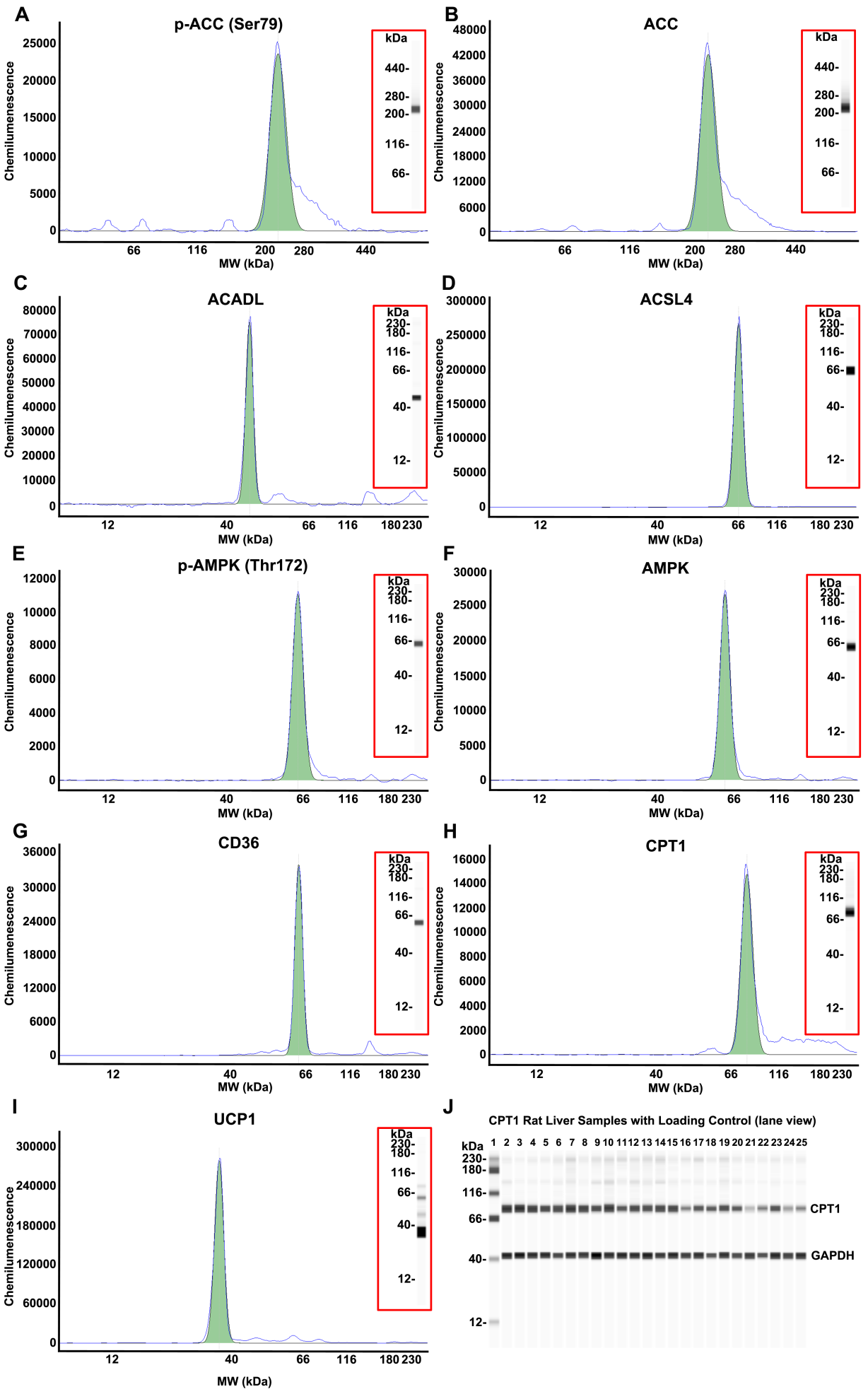


**Supplementary Figure S1.** Antibody quality control data. Rat liver lysates were used for all antibodies except UCP1, which was done using rat interscapular brown fat lysate. Concentration for all lysates was 0.2 mg/ml and all primary antibodies were diluted 1:50. (**A-I**) Image within the red box inset is the lane view or virtual western blot-like image for each electropherogram as generated by the Compass software, ver. 3.1.7 (ProteinSimple, San Jose, CA) and antibodies were screened on pooled samples without loading controls to assess purity; (**J**) Lane view of rat liver samples probed with CPT1 and GAPDH loading control, lanes 2-25 (ladder, lane 1).
