# Supplementary material for: Beneficial Effects of Common Bean on Adiposity and Lipid Metabolism
Source: Nutrients. 2017 Sep 9;9(9):998. doi: 10.3390/nu9090998 (PMC5622758; doi:10.3390/nu9090998)
Supplement: Supplementary file 1 [file nutrients-09-00998-s001.zip › Supplementary Figure S2-Adipocyte Analysis.docx]

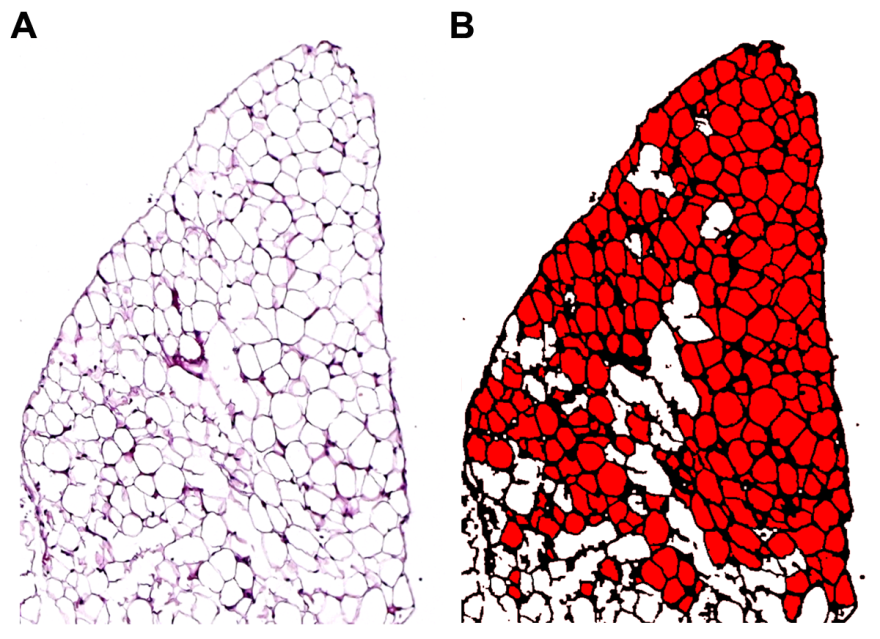


**Supplementary Figure S2.** Adipocyte image analysis. (**A**) Raw image of H&E stained visceral (parametrial) fat, x 100 magnification; (**B**) Adipocytes marked for image analysis (red) versus unselected areas (white).
