# Supplementary material for: Beneficial Effects of Common Bean on Adiposity and Lipid Metabolism
Source: Nutrients. 2017 Sep 9;9(9):998. doi: 10.3390/nu9090998 (PMC5622758; doi:10.3390/nu9090998)
Supplement: Supplementary file 1 [file nutrients-09-00998-s001.zip › Supplementary Figure S3-UCP1.docx]

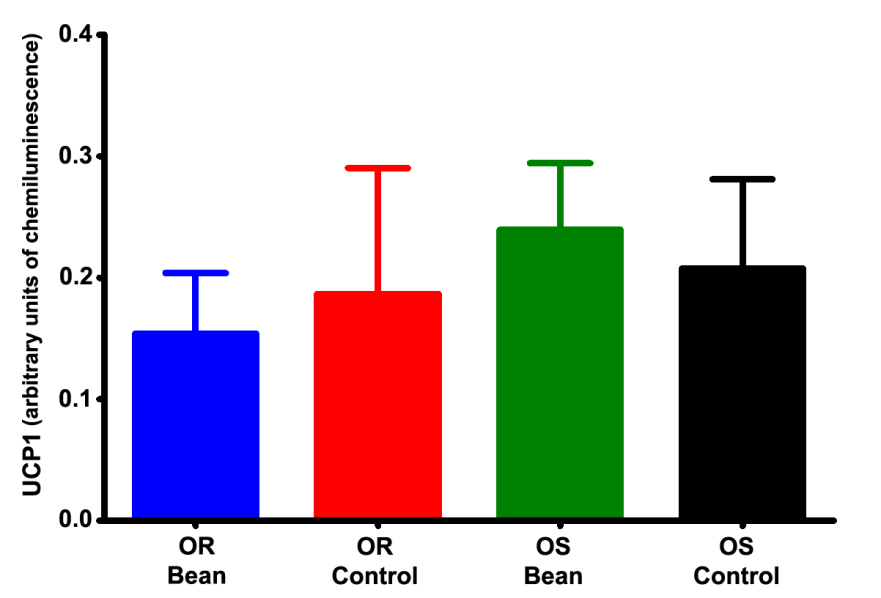


**Supplementary Figure S3.** UCP1 expression in visceral (parametrial) fat (peak height normalized to loading control); ANOVA: strain *p* = 0.478, diet *p* = 0.997, interaction *p* = 0.665.
